# Latest Considerations in Diagnosis and Treatment of Appendicitis During Pregnancy

**DOI:** 10.5811/cpcem.2018.1.36218

**Published:** 2018-03-14

**Authors:** Shahram Lotfipour, Max Jason, Vincent J. Liu, Mohammad Helmy, Wirachin Hoonpongsimanont, C. Eric McCoy, Bharath Chakravarthy

**Affiliations:** * University of California, Irvine, Department of Emergency Medicine, Orange, California; † Taipei Medical University, College of Medicine, Taipei, Taiwan; ‡ University of California, Irvine, Department of Radiological Sciences, Orange, California

## Abstract

Pregnancy can obscure signs and symptoms of acute appendicitis, making diagnosis challenging. Furthermore, avoiding radiation-based imaging due to fetal risk limits the diagnostic options clinicians have. Once appendicitis has been diagnosed, performing appendectomies has been the more commonly accepted course of action, but conservative, nonsurgical approaches are now being considered. This report describes the latest recommendations from different fields and organizations for the diagnosis and treatment of appendicitis during pregnancy.

## INTRODUCTION

Appendicitis among pregnant women is the most common cause of non-gynecological or obstetric-related emergency surgeries.^1^ Appendicitis occurs in 0.05% to 0.07% of pregnancies with the highest frequency of cases occurring during the second trimester of pregnancy.^2^ Pregnant women are more likely to experience perforation of the appendix, with rates as high as 55%, compared with 4% – 19% in the general population.^2^ During pregnancy, symptoms of appendicitis may appear seemingly normal and anatomical changes may obscure classic signs, thereby confounding the diagnosis of appendicitis.^1^ In addition to detection challenges, incorrect diagnoses may result in negative appendectomies, putting fetuses at unnecessary risk of spontaneous abortions and premature deliveries.^3^

When a non-pregnant patient displays symptoms indicative of appendicitis, transabdominal sonography and computed tomography (CT) are typically the imaging modalities of choice. Ultrasound (US) serves as a quick and readily available initial tool but may be inconclusive due to factors such as operator skill, patient physique, and intrinsic resolution. In contrast, CT has a sensitivity of 91% and specificity of 90% in diagnosing appendicitis.^4^ Although CT outperforms US in accuracy, in order to reduce fetal risk of complications due to radiation exposure, radiologists’ preferences are shifting toward low-dose CT and, predominantly, magnetic resonance imaging (MRI) when an initial US is non-diagnostic.^5^,^6^ It is not uncommon that MRI would not be available in an ED, or have limited availability during the night, which would necessitate an early decision for patient transfer to obtain higher level-of-care diagnostic studies.

In terms of treatment, performing an appendectomy is the current treatment of choice. Recent research explores a conservative, non-operative, antibiotic treatment approach as an option, but this practice is not widely accepted and may lead to recurrent appendicitis.^7^ We describe considerations in the diagnosis and treatment of suspected appendicitis in a pregnant woman with a history of lupus, kidney disease, and hypertension. We also describe her care in respect to the latest guidelines associated with management of acute appendicitis during pregnancy. Because the symptoms of appendicitis can be similar to those of pregnancy, diagnosis can be challenging given the need to avoid radiation. This report details up-to-date information regarding maternal treatment recommendations. We address the diagnostic and treatment challenges clinicians face with such a patient presentation.

## CASE REPORT

A 23-year-old female at a gestational age of 13 weeks and three days presented to the emergency department (ED) with acute abdominal pain and dyspnea. The patient awakened at 5:00 am with sudden epigastric pain that intensified and became more diffuse by the time she presented at 8:33 am. The patient had a history of lupus with associated stage I kidney disease and hypertension. She intermittently took steroids for lupus flares but had no history of corresponding bowel or abdominal symptoms.

A physical exam showed that the patient had a temperature of 36.7° Celsius, heart rate of 93 beats per minute, blood pressure of 123/93 millimeters of mercury, respiratory rate of 18 breaths per minute, and a blood oxygen saturation of 100%. The patient was in mild distress due to pain, which had increased from 6/10 to 8/10 since its onset. She denied similar pain during her single previous pregnancy. She had two episodes of emesis with light yellow vomitus since she woke up. The patient exhibited normal bowel sounds, no tenderness at McBurney’s point, no suprapubic tenderness, and no costovertebral angle tenderness. She described pain around her umbilicus, which she believed to be different in character and location from her lupus flares, which are often characterized by migraines and nausea.

**Image 1:**
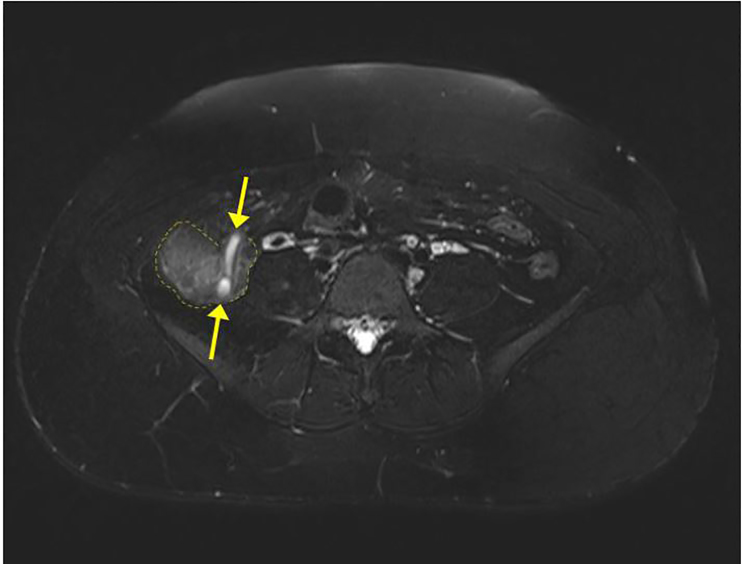
Axial T2-weighted magnetic resonance image demonstrates a dilated appendix (demarcated by yellow arrows) with increased signal of surrounding fat indicating inflammation (outlined by dashed yellow lines).

**Image 2:**
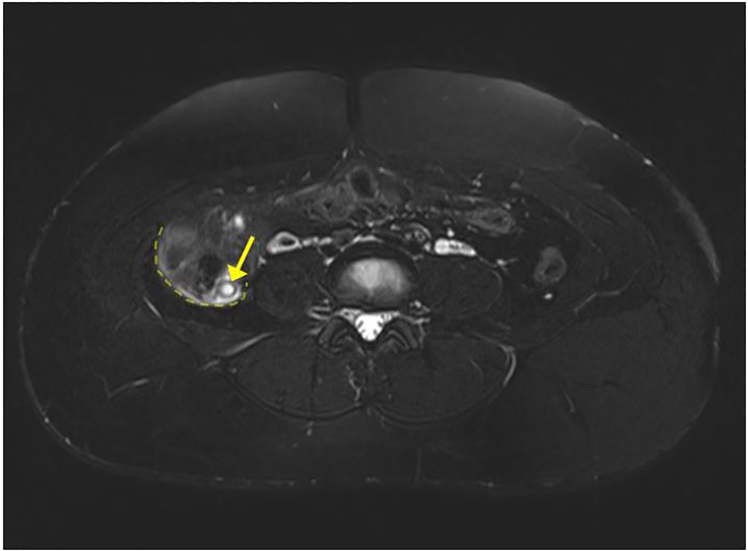
Axial T2-weighted magnetic resonance image demonstrates a dilated appendiceal base (yellow arrow) measuring up to 1 cm in diameter with mural thickening, periappendiceal fluid, and increased signal of surrounding fat indicating inflammation (outlined by dashed yellow line).

US imaging was performed on the right lower quadrant but the appendix was not visualized. US did successfully confirm a live intrauterine pregnancy. A transvaginal exam of the pelvis showed normal ovaries, and fetal biometry measurements were consistent with dates. After morphine administration, the patient’s pain decreased and became localized in the right lower quadrant. A surgeon was then consulted and recommended MRI of the abdomen and pelvis without contrast to evaluate the appendix. The MRI of the abdomen showed borderline appendicitis with stranding, minor wall thickening, free fluid, but no abscess (images 1–2). The patient was diagnosed with acute appendicitis and was admitted to the surgical service. The surgical team successfully performed a laparoscopic appendectomy and she was discharged the following day. The pathological report confirmed the initial diagnosis of acute appendicitis after microscopy of the appendix.

## DISCUSSION

Appendicitis manifests with similar symptoms as those of pregnancy; though rare, it affects approximately one in 1,500 pregnancies. It is the most common cause of emergency non-gynecologic and non-obstetrical surgery in pregnant women.^8^ Appendicitis is difficult to identify in pregnant patients due to the patient characteristics that obscure otherwise-classic signs or symptoms. Major symptoms include vomiting, anorexia, nausea, pyrexia, tachycardia, and lower right quadrant pain.^9^ The appendix may shift upwards during pregnancy and patients may experience pain in the right upper quadrant or right flank.^8^

**CPC-EM Capsule**

What do we already know about this clinical entity?

Appendicitis is the most common cause of non-gynecological or obstetric-related emergency surgeries.

What makes this presentation of disease reportable?

This presentation is reportable given the prevalence of appendicitis and the degree of vigilance required to ensure proper diagnosis and treatment.

What is the major learning point?

Magnetic resonance imaging is the latest recommended diagnostic imaging modality. Appendectomies remain the preferred therapy over non-operative approaches.

How might this improve emergency medicine practice?

Increasing awareness of the possibility of appendicitis among the pregnant population in which symptoms can be obscured, could improve emergency medicine practice.

Physical exam techniques conventionally used in diagnosis, such as Rovsing’s and psoas signs, are ineffective in the case of pregnant patients.^10^ In addition, leukocytosis is not a reliable metric for pregnant patients as it occurs physiologically during pregnancy.^9^,^10^ Pyuria is observed in 10%–20% of patients and may be concurrent with asymptomatic or symptomatic bacteriuria found in the pregnant population.^11^ It is important to consider other gastrointestinal, obstetric, and gynecological diagnoses that present with similar symptoms. Non-imaging scoring systems are useful diagnostic tools to stratify patients with suspected appendicitis. The Alvarado score is one that has been validated and a score cut-off of five can be useful in ruling out the diagnosis of appendicitis. 12,13

For non-pregnant patients, CT has been demonstrated to be the most accurate method for diagnosis. Contrast-enhanced CTs have diagnostic accuracy ranging from 91%–95% with specificity of 90%–95%. Unfortunately, a standard CT exposes a pregnant woman and her fetus to undesired radiation. In the case of a pregnant patient, the American College of Radiology recommends initial imaging using US, which offers 67%–86% sensitivity and 76%–88% specificity when imaging non-pregnant patients.^6^ Use of US is often operator-dependent, and identification of appendicitis in pregnant women can easily be hampered by bowel gas and obesity.^14^ For patients in their late second or third trimester, it is recommended that they be placed in the left posterior oblique or left lateral decubitus position to allow displacement of the enlarged uterus and facilitate use of graded compression techniques.^15^

In a retrospective study of pregnant patients, US was found to be effective in visualizing the appendix only in 7% of cases with 18% sensitivity and 99% specificity.^16^ If an US diagnosis of acute appendicitis is indeterminate in a pregnant patient, MRI should be used. MRI visualizes the appendix with 100% sensitivity and 98% specificity.^16^ MRI does not emit ionizing radiation and has no known adverse effects on either the mother or fetus.^17^,^18^ Other studies have shown that MRI has a positive predictive value of 90.4% and negative predictive value of 99.5%, if the appendix can be identified.^6^ MRI is the current gold standard for accurately diagnosing appendicitis in pregnant patients after an inconclusive US.^6^

Pregnancy adds an additional layer of treatment challenges when addressing appendicitis. Accurate diagnoses are important for pregnant women exhibiting abdominal pain because of possible complications stemming from either a delayed or negative appendectomy. False positive diagnoses and subsequent surgeries put pregnant women at unnecessary risk. A large retrospective study demonstrated evidence of a fetal loss rate of 4% and early delivery rate of 10% for negative appendectomies.^3^ Given the risks associated with delayed diagnosis, the current practice when acute appendicitis is highly suspected is to perform an immediate appendectomy because any delay in surgery could lead to a ruptured appendix and increased fetal mortality.^11^

Though conservative treatment of appendicitis with antibiotics has recently gained attention as an alternative treatment option, Salminen et al. were unable to demonstrate non-inferiority compared to appendectomies among patients 18–60 years old with uncomplicated acute appendicitis. 19 A nonsurgical approach may reduce complication rates, but efficacy of surgery is currently still significantly higher.^20^ Therefore, currently both open and laparoscopic appendectomies are considered appropriate surgical techniques; however, some studies have shown that laparoscopic interventions should not be performed in the third trimester.^21^

## CONCLUSION

When a pregnant patient arrives at the ED with symptoms indicative of appendicitis, ultrasound is recommended as the first line of diagnosis. However, due to the difficulty in viewing the appendix in a pregnant patient using ultrasound, MRI is the best tool for diagnosis. MRI does not present the same radiation risk to the fetus as CT and provides comparable diagnostic power. It is not uncommon that MRI would not be available in an ED, or have limited availability during the night, which would necessitate an early decision for patient transfer to obtain higher level-of-care diagnostic studies. It is important to quickly ascertain a correct diagnosis as delayed appendectomies can lead to ruptures and subsequently higher fetal mortality rates. Standard of care after acute appendicitis is diagnosed in a pregnant patient is surgical consultation for an emergency appendectomy, as efficacy and safety of non-operative management with antibiotics in pregnant patients remains to be elucidated.

Documented patient informed consent and/or Institutional Review Board approval has been obtained and filed for publication of this case report.
